# Brain rewiring during development: A comparative analysis of larval and adult *Drosophila melanogaster* connectomes

**DOI:** 10.1162/NETN.a.26

**Published:** 2025-11-20

**Authors:** Prateek Yadav, Pramod Shinde, Aradhana Singh

**Affiliations:** Department of Biology, Indian Institute of Science Education and Research, Tirupati, India; La Jolla Institute for Immunology, La Jolla, CA, USA; Department of Physics, Indian Institute of Science Education and Research, Tirupati, India

**Keywords:** Connectomics, Fruit fly, Neurodevelopment, Core-periphery structure, Rich club

## Abstract

The brain’s ability to undergo complex rewiring during development is a fascinating aspect of neuroscience. This study conducts a detailed comparison of *Drosophila melanogaster*’s brain networks during larval and adult stages, revealing significant changes in neuronal wiring throughout development. The larval brain network exhibits a degree distribution that fits firmly to a Weibull model. In contrast, the sparser adult brain network follows a power-law distribution, with the out-degree exponent lying in the scale-free regime and the in-degree exponent close to it. This shift toward a scale-free pattern likely reflects an adaptation to enhance robustness against failures while minimizing costs associated with reduced density during development. We also observed alterations in the structural core in relation to cell composition and topological influence. The structural core of the larva comprises neurons in the mushroom body, while neurons in the antennal lobe form the core of the adult fly brain. Furthermore, the larval network solely shows a rich club organization of which the structural core is also a part. Analysis of connectivity, rich club, and network measures reveals that the shift in the core results from a reduction in the centrality of mushroom body neurons following metamorphosis. This work stands as a step forward in understanding the rewiring of brain networks across the life stages of *D. melanogaster*.

## Author Summary

We investigate developmental changes in neuronal rewiring by analyzing the complete connectomes of larval and adult fruit fly brains. The adult brain shows a shift in connectivity, with its out-degree following a scale-free distribution. Moreover, the core of the network, the integrative hub, shifts from mushroom body neurons in the larval to antennal lobe neurons in the adult brain. Notably, the larval core is embedded within the rich club—a dense, influential neuronal group—revealing a hierarchical structure where the core neurons form a “rich club within the rich club.” This configuration disappears in the adult phase, indicating developmental reorganization and functional reallocation during the development of the fruit fly.

## INTRODUCTION

Brain connectomics is the comprehensive study of neural connectivity patterns, encompassing both structural and functional relationships on various spatial scales within the nervous system (Behrens & Sporns, 2012; Sporns, Tononi, & Kötter, 2005). The mapping of the brain assigning the links between the different components of the brain including neurons, or different brain regions through axons, fiber tracts, or temporally correlated activation (Fornito, Zalesky, & Bullmore, 2016) that mainly relies on experimental techniques such as diffusion-weighted magnetic resonance imaging, functional magnetic resonance imaging (fMRI), electroencephalography (EEG) for macroscale studies, serial sectioning of nervous tissue, and electron microscopy for microscale studies (Yap, Wu, & Shen, 2010). To date, extensive brain mapping has been achieved that reveals connectivity patterns at the cellular level for only a few species, one of which is *Drosophila melanogaster*, commonly known as the fruit fly. Drosophila is an exemplary model organism for connectomics research due to its extensive use in genetics and molecular biology. The substantial knowledge and comprehensive experimental protocols associated with *D. melanogaster* biology makes it a valuable resource for connectomics investigations. Additionally, *D. melanogaster* exhibits a diverse repertoire of well-characterized behaviors, further improving its suitability as a model organism for studying the neural circuitry underlying behavior and cognition (Jennings, 2011; Sokolowski, 2001).

The complete metamorphosis of higher insects, including *Drosophila*, is not only fascinating but also shrouded in mysteries, particularly those related to the nervous system. The transition from the larval stage to a morphologically and physiologically distinct adult form in *Drosophila* necessitates complex alterations in the nervous system (Veverytsa & Allan, 2012; Yaniv & Schuldiner, 2016). Certain types of neurons associated with metamorphosis, mating, hormonal regulation, and so forth, are only found in the adult brain (Lin et al., 2024; Truman, Price, Miyares, & Lee, 2023; Winding et al., 2023). In addition, the moonwalker descending neuron circuit and associated Pair1 interneurons responsible for locomotion in the larval stage undergo remodeling during metamorphosis (Lee & Doe, 2021). As the brain performs tasks through the coordination between the different neuronal cells, studying its rewiring is useful in understanding how neurological structures translate to function (Cheong et al., 2024). With the generation of the complete connectomes of the larval and adult forms of the fruit fly (Lin et al., 2024; Winding et al., 2023), we had the opportunity to compare the two brain networks and explore the insights that network science can offer through such a comparison.

Furthermore, in various real-world networks, the central cores exist within a broader core-periphery organization, where a densely connected core integrates with a more weakly connected periphery—often shaped by resource constraints and connection costs (Csermely, London, Wu, & Uzzi, 2013). The dense interconnectivity within the core enhances network resilience by promoting degeneracy, which allows different components to perform similar functions (Kitano, 2004; Peixoto & Bornholdt, 2012). While degeneracy helps the network recover from random node failures, a core-periphery structure can make the system more susceptible to targeted attacks. These contrasting roles of robustness and vulnerability are key aspects of the core-periphery organization, making it crucial to determine whether brain networks exhibit such an organizational pattern (Bullmore & Sporns, 2012). Indeed, encouraging findings in recent times highlight the potential significance of core structures in brain networks, motivating our study. At the same time, their functional and behavioral correlates remain an emerging area of exploration. A k-core decomposition analysis carried out separately for in- and out-degree revealed that the k-core for in-links consisted predominantly of motor neurons while the k-core of out-links primarily comprised sensory neurons in the nervous systems of *C. elegans* (Chatterjee & Sinha, 2007). In the human cortex, the core regions are associated with consciousness, providing a platform for its emergence (Lahav et al., 2016). A study of human functional brain networks using fMRI revealed that the k-core comprises the brain regions active in the brain’s subliminal state (Lucini, Del Ferraro, Sigman, & Makse, 2019). Similarly, rich clubs (RCs) have been reported in the connectomes of *C. elegans* (Towlson, Vértes, Ahnert, Schafer, & Bullmore, 2013), cat (de Reus & van den Heuvel, 2013; Zamora-López, Zhou, & Kurths, 2010), and mouse (Oh et al., 2014). The disruption of RC connectivity has been linked to various neurological and pathological disorders, providing insights into their underlying pathologies (Buckner et al., 2009; Collin, Kahn, De Reus, Cahn, & Van Den Heuvel, 2014; Van Den Heuvel et al., 2013). Both k-core decomposition and RC analyses offer a mesoscopic view of how influential nodes interact with each other and impact the rest of the network. While k-core analyses typically focus on undirected relationships by measuring the degree of nodes alone, the D-core decomposition method (Giatsidis, Thilikos, & Vazirgiannis, 2013), which is the directed counterpart of the k-core, incorporates both in-degrees (k) and out-degrees (l) based on (k, l) restrictions. Given that chemical synapses in the brain mediate directional communication between neurons, we primarily relied on the D-core analysis to study the core.

Our study aims to develop a network-based framework to enhance the system-level understanding of the nervous system metamorphosis in *Drosophila*. Notably, we aspire to compare and contrast neuronal connectivity between larval and adult *Drosophila* brain networks based on their degree distribution and the core decomposition. To achieve this, we analyze two publicly available datasets: one providing the whole-brain connectome of a female fruit fly larva (first instar) (Winding et al., 2023) and another encompassing the entire brain of an adult female fruit fly (Flywire dataset v630) (Dorkenwald et al., 2023; Schlegel et al., 2023).

We identify structural similarities between the two networks, including modularity, high clustering, and the absence of strong connectivity. However, we also observe differences, for instance, the larval brain is weakly connected, whereas the adult brain is not. A directed graph is considered weakly connected if, when the direction of its edges is disregarded, the graph remains connected. In contrast, a directed graph is strongly connected if every vertex can be reached from every other vertex by following the direction of the edges. Additionally, we find that the degree distribution of the larval brain follows a stretched exponential pattern and fits well with the Weibull distribution (Newman, 2018), whereas the adult brain exhibits a strong adherence to a power-law distribution in both the in-degree and out-degree distributions, with their exponents being within or very close to the scale-free regime. Furthermore, we find that the core of the brain network undergoes remodeling during metamorphosis. In the larval stage, the core consists of mushroom body neurons (MBNs), which are involved in learning and memory. These core nodes also form the RC. In contrast, the core of the adult brain comprises antennal lobe neurons (ALNs), which do not form an RC. At each developmental stage, all core neurons are confined to a single neuropil. However, not all neurons within that neuropil form the core, indicating an internal hierarchy within a neuropil where specific cells hold more influential roles within the network. The transition of the core from MBNs to ALNs is also reflected in the relatively lower betweenness centrality values of MBNs compared with the network average in the adult phase, suggesting the occurrence of significant developmental changes in the brain as the organism matures.

## RESULTS

### Distinct Structural Properties Across *Drosophila* Developmental Stages

The structural properties of the adult and larval *Drosophila* developmental stages have been assessed, disclosing notable differences between their brain networks (Table 1). The adult brain is much larger than the larval brain, with over 40 times the number of nodes (neurons, *N*; see Table 1). This difference in size could be attributed to the adult brain’s need to support much more complex behaviors, such as foraging, navigation, and mating (Anderson, Scott, & Dukas, 2016). The larval brain network exhibits a much higher connection density (*d*), which makes it weakly connected (Table 1); the same is not valid for the adult brain.

**Table 1.** Network properties of adult and larval connectome

Network	*N*	*d*	〈*C*〉_*n*_	*Q*_*n*_	SC	WC	*r*_*s*_	〈*btw*〉_MBNs_
Adult	124,891	0.0002	24.13	420.79	False	False	0.948	0.68
Larval	2,952	0.01	6.44	166.60	False	True	0.894	1.33

Here, *N*, *d*, 〈*C*〉_*n*_, *Q*_*n*_, SC, WC, *r*_*s*_, and 〈*btw*〉_MBNs_ represent the number of nodes, connection density, the average clustering coefficient normalized by degree preserved null models, normalized modularity of Louvain partitions obtained after consensus community detection across different resolutions, state of being strongly connected, state of being weakly connected, Spearman correlation between the unweighted and weighted degrees, and the average betweenness centrality of the MBNs as stated in Equation 1, respectively. Regarding parameter normalization, we used 100 null models for calculating the clustering of the larval and 20 for modularity. In the case of the adult network, we employed 10 null models for calculating clustering and two for modularity. Pertaining to the adult network, we used the largest strongly connected component for consensus community detection and, thus, modularity calculations.

Moreover, these basic network properties also reflect similarities between the larval and the adult brain networks. Both networks are not strongly connected, as shown in (Table 1). A directed network is strongly connected if there is a path from every node to every other node. Notably, both the connectomes exhibit a strong Spearman correlation between the unweighted and weighted degrees (*r*_*s*_; Table 1). Additionally, both networks exhibit higher modularity values (*Q*) compared with the corresponding degree-preserved random networks, suggesting a strong modular structure with well-partitioned communities that are densely connected within themselves but less connected among each other. However, the adult brain displays a higher normalized modularity (*Q*_*n*_; Table 1) and clustering than the larval brain, indicating that the adult brain network comprises densely connected components performing more independent specialized functions than the larval network. This finding is consistent with another study that found an increase in modularity as the brain network of *C. elegans* develops (Witvliet et al., 2021). We further examined the structures of both networks in detail.

**Figure 1.**
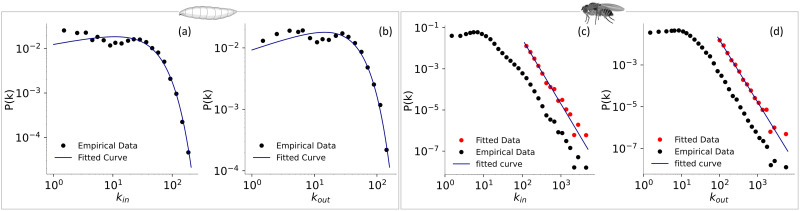
The in-degree and out-degree distribution of the larval and adult brain networks on a log–log scale. Subplots (A) and (B) show the in-degree (*k*_in_) and out-degree (*k*_out_) distributions of the larval connectome, while subplots (C) and (D) show the corresponding distributions for the adult brain network. In subplots (A) and (B) for the larval brain, the distributions are fitted with the Weibull distribution, while in subplots (C) and (D) for the adult brain, they are fitted with the power-law distribution using the maximum likelihood method mentioned in the Methods section. In subplots (C) and (D), the black points represent the probability distribution function for the empirical data, while the red points represent the probability distribution function of the fitted data (which includes degree values ≥ *x*_min_). Further details on the minimum degree fitting with the power-law are provided in Supporting Information Table S1.

To gain deeper insights into the organization of connections within brain networks, we further analyze the degree distribution of these networks. We find an intriguing observation regarding the degree distribution for both in-degree and out-degree in adult and larval brain networks. The degree distribution exhibits two distinct behaviors in the initial and tail segments. In the larval degree distribution, the initial segment displays a noticeable plateau, representing a significant proportion of the overall distribution. In contrast, such a distinction is less pronounced in the adult network (Figure 1). This observation suggests unique structural characteristics in the larval stage compared with the adult stage. The presence of a fat tail in the degree distribution of both networks indicates the existence of hubs. Previous studies have demonstrated that many real-world networks, such as the frequency distribution of earthquake magnitudes, word frequency, citations, telephone calls, the intensity of wars, and the net worth of wealthy Americans, exhibit hub-like structures, as indicated by the fat tail in their degree distributions (Newman, 2005). These fat tail distributions often exhibit a power-law behavior, where the probability function for the degree distribution is given by the form *P*(*k*) ∝ *k*^−*γ*^, where *P*(*k*) refers to the probability of finding a node with degree *k* in the network and *γ* is the power-law exponent. For the exponent 2 < *γ* < 3, the scale-free property emerges as the second moment of the degree distribution diverges (Newman, 2005). Consequently, we decided to examine whether the degree distributions of fly connectomes fit a power-law.

### Degree Distribution and Network Growth in Larval and Adult Brain Networks

We observe that the degree distribution of the larval brain network follows a stretched exponential pattern, with only a small proportion of data fitting a power-law distribution (Supporting Information Figure S1). Both in- and out-degree fit well with the Weibull distribution (Figures 1A, B). The Weibull distribution is a mixture of the exponential and the power-law, and therefore, the probability function for the Weibull distribution has two parameters and is given as *P*(*k*) ∝ *k*^*β*−1^*e*^−*λk^β^*^ (Clauset, Shalizi, & Newman, 2009). For the parameter *β* = 1, it becomes pure exponential. We use maximum likelihood estimation (MLE), as mentioned in the Methods section, for this fitting. The entire range of the in-/out-degree of the larval brain network fits well with the Weibull distribution, confirmed by the low Kolmogorov–Smirnov distance values 0.0396 and 0.0388, respectively. The distribution parameters *λ* and *β* are determined to be 0.0247 and 1.2409 for in-degree and 0.0244 and 1.3348 for the out-degree.

The Weibull distribution has also been shown to better fit the degree distribution of the functional human brain network (Zhang et al., 2021). However, as the fruit fly matures into a fully developed adult, and the brain network grows, the in- and out-degree distribution fits poorly with the Weibull distribution (Supporting Information Figure S1). Instead, both the in- and out-degree of the adult brain network fit well with the power-law distribution, with most of the data falling on the straight line, fitting the distribution on the log–log plot (Figures 1C and 1D). Detailed statistics of this fitting can be found in Supporting Information Table S1. It is noticeable that the exponent of the power-law for the out-degree of the adult brain is in the scale-free regime (*α* = 2.96), and for the in-degree, it is very close to the scale-free regime (*α* = 3.15), indicating the existence of a scale-free nature in this network, which is missing in the larval network. This suggests that as the fruit fly’s brain develops, the scale-free nature of the network may emerge due to various factors, potentially contributing to network robustness (Supporting Information Figure S7).

Adaptation to the scale-free topology could also represent an evolutionary optimization for energy efficiency, constrained by the need to maintain adequate information processing. Adherence to a power-law in the adult brain networks indicates a higher prevalence of putative hubs. In contrast, in the larval network, only high-degree neurons follow the power-law, suggesting the presence of fewer putative hubs than in the adult network. Such patterns likely reflect underlying evolutionary processes that influence the formation and stabilization of adult brain networks.

To delve deeper into these network differences, we employ RC and D-core analyses, which provide enhanced insights into the intricate organization of connections among the high-degree neurons and the core structures within each network, highlighting their hierarchical and functional organization.

### D-Core Decomposition and RC Analyses

**Figure 2.**
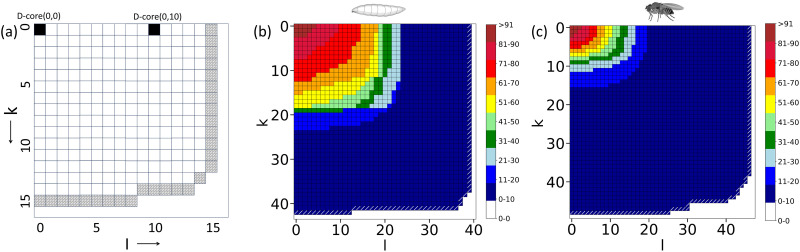
D-core decomposition of the networks. (A) A schematic of the D-core matrix with the frontier cells filled with diagonal brick patterns. Each (k, l) cell represents a D-core (k, l). Here, k stands for in-degree, and l stands for out-degree in the decomposition process. (B) and (C) plot the D-core matrix of the larval and adult fruit fly connectomes, respectively. The frontier D-cores in these plots are highlighted by white-colored oblique lines. The color bar indicates the percentage of the neuronal count in the different D-cores. Please note that in these plots, the values k and l denote the degree of the nodes in D-core in the decomposition process, not the actual degrees of the nodes in the network.

The studied brain networks are directed, with each node having two types of neighbors: one acting as the source (providing information to it) and the other as a sink (receiving information from it). Therefore, a two-dimensional analog of the k-core decomposition, known as the D-core decomposition (Giatsidis et al., 2013), is necessary. Following the approach detailed in the Methods section, we obtain the nodes belonging to the frontier D-cores for both the larval and adult connectomes. Although the adult brain is significantly larger than the larval brain, it loses connections more rapidly during the core decomposition process than the larval brain network because of the sparsity of connections. As depicted in Figures 2B and 2C, in the D-core (10, 8), the adult brain loses almost 80% of its neurons, whereas in the same D-core, the larval brain loses approximately 20% of its neurons. Additionally, the heterogeneity of cell types in the frontier D-cores is similar for both brain networks (Supporting Information Figure S2). These frontier D-cores, being the innermost D-cores and having highly interconnected neurons, serve as the core; thus, henceforth, we refer to them as the network’s core. In the following section, we examine the compositions of the core neurons and their neighbors in detail to gain a comparative understanding of the core of the larval and adult brain networks.

#### The larval core is composed of MBNs.

**Figure 3.**
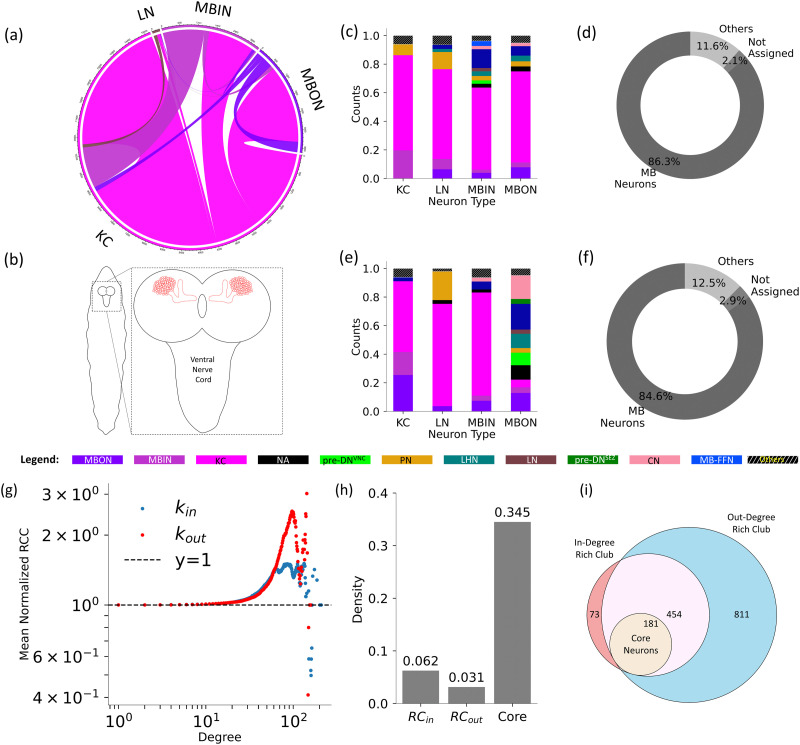
Analysis of the core of the larval connectome. The chord diagram in (A) plots the neuron types present in the core and the connections among them. (B) displays a schematic diagram of the larval brain, with the mushroom bodies drawn in red. In (C) and (E), the normalized cell type composition of predecessors and successors of the different types of core neurons is plotted. Pie charts in (D) and (F) summarize the type composition of predecessors and successors of all core neurons. (G) plots mean normalized *RCC* versus degree; bars in (H) plot connection density of the in-degree RC, out-degree RC, and core; and (I) again presents the statistics of the in-degree and out-degree rich club neurons.

In the larval brain, the core consists of 181 neurons, including 114 Kenyon cells (KCs), 44 mushroom body output neurons (MBONs), 21 mushroom body input neurons (MBINs), and two local interneurons of the anterior paired lateral cell (Figure 3A). All of these neurons not only belong to the mushroom body (MB), a structure traditionally recognized as a center for olfactory memory, but also implicated in a variety of other behavioral tasks, including odor preference, taste preference, and associative learning between odor and taste (Pauls, Selcho, Gendre, Stocker, & Thum, 2010; Saumweber et al., 2018). Each of the neuron types found in the core has a particular role in the functioning of the memory circuit. KCs, located in the calyx of the MB, obtain olfactory sensory information from the sense organs (Thum & Gerber, 2019; Vosshall & Stocker, 2007) and send outputs to other KCs, MBINs, and MBONs (Eichler et al., 2017; Thum & Gerber, 2019). On the other hand, the gustatory information from the same chemosensory apparatus that includes the olfactory sensory system is relayed to the MBINs (Thum & Gerber, 2019; Vosshall & Stocker, 2007). MBINs establish synaptic connections at both the presynaptic and postsynaptic terminals of multiple KC-to-MBON synapses (Eichler et al., 2017). They are composed of Dopaminergic Neurons, octopaminergic neurons, and a few neurons whose neurotransmitters have not been identified yet (Thum & Gerber, 2019). MBONs are output neurons that are responsible for generating motor output based on the olfactory and gustatory information (Thum & Gerber, 2019). They receive input from KCs, MBINs, other MBONs, and non-MB neurons (Eichler et al., 2017). We also perform k-core decomposition analyses, as explained in the Methods section, which reveals the presence of MB neurons in the central core (Supporting Information Figure S6). These neurons are a subset of the frontier D-core neurons.

Furthermore, we explore which neuronal cell types are directly connected with the core neurons, as these neurons act as a bridge between the other neurons and the core. Neurons that are postsynaptic to frontier neurons are termed their successors, while those presynaptic to frontier neurons are termed their predecessors.

All four types of core neurons are mostly connected to the KC neurons, followed by MBIN neurons, and then by MBON neurons, which are the MB neurons (Figure 3C). Additionally, there are connections to MB feedback neurons (MB FBNs) and Mushroom Body Feedforward Neurons (MB-FFNs), all of which belong to the same neuropil as the frontier neurons, thus demonstrating homogeneous connectivity. In total, these MB neurons account for 86.3% of the neighbors of the core, indicating that the nearest neighbors of core neurons are similar types of neurons. The interspecific types include projection neurons (PNs), convergence neurons (CNs), pre-DNs^VNC^ (Predescending Neuron to the Ventral Nerve Cord), and pre-DNs^SEZ^ (Predescending Neuron to the Subesophageal Zone). This information is well summarized by the pie chart in Figure 3D. The successors of the core also mostly exhibit a similar type of composition pattern as the predecessors. However, the successors of the core differ from predecessors in having a greater type diversity among the neighbors of the core MBON neurons (Figure 3E), which is consistent with their well-established role in generating behavioral responses.

Furthermore, we perform a RC analysis to gain deeper insight into the core of the larval brain. This analysis focuses on a group of high-degree nodes more interconnected than their corresponding random surrogates. We calculated the rich club coefficient (*RCC*) as defined in the Methods section for the corresponding 100 degree preserved random networks, and then normalized the *RCC* obtained for the larval brain network using these values. This normalized *RCC* is plotted in Figure 3G. We find that the larval connectome shows prominent RC organization for both in- and out-degree (Figure 3G). The density of the in-degree RC neurons and out-degree RC neurons is 0.062 and 0.031, respectively, which is six times and three times, respectively, the whole network’s density (Figure 3H). Further analysis indicates that all neurons within the frontier D-cores are also part of the RC. The density of these core neurons is 0.345, approximately 30 times greater than that of the entire network (Figure 3H). This significant increase in connectivity within the core compared with other RC neurons suggests that the core represents a more intensely connected subgroup. In essence, it can be regarded as the “rich club of the rich club,” emphasizing its critical role in network communication and function (Figure 3G).

Moreover, considering in-degree, the top 24% of nodes have more connections than their degree-preserved random surrogates. Similarly, considering the out-degree, the top 49% of the nodes (for obtaining top nodes, ties were broken by eigenvector centrality values) are more densely connected than their random surrogates. These RC cohorts include 708 and 1,446 nodes, respectively, for in- and out-degrees (as indicated by the normalized *RCC* values being greater than 1 in Figure 3G). Both cohorts contain a wide range of cell types: The former lacks only three cell types—RGN (Ring Gland Neuron), sensory, and ascending—while the latter excludes only RGN-type neurons. This indicates a strong connection between most cell types in the larval brain. Out of these RC neurons, there are 181 core neurons, including 114 KCs, 44 MBONs, 21 MBINs, and 2 local interneurons of the anterior paired lateral cell.

#### The core of the adult fly brain is composed of antennal lobe (AL) neurons.

The Flywire dataset organizes neurons into multiple hierarchical levels, and we have determined that the “class” level is the most suitable for meaningful comparisons with the larval connectome, where “cell type” annotations are used. Henceforth, all type-based analyses of the adult connectome utilize the “class” annotation.

**Figure 4.**
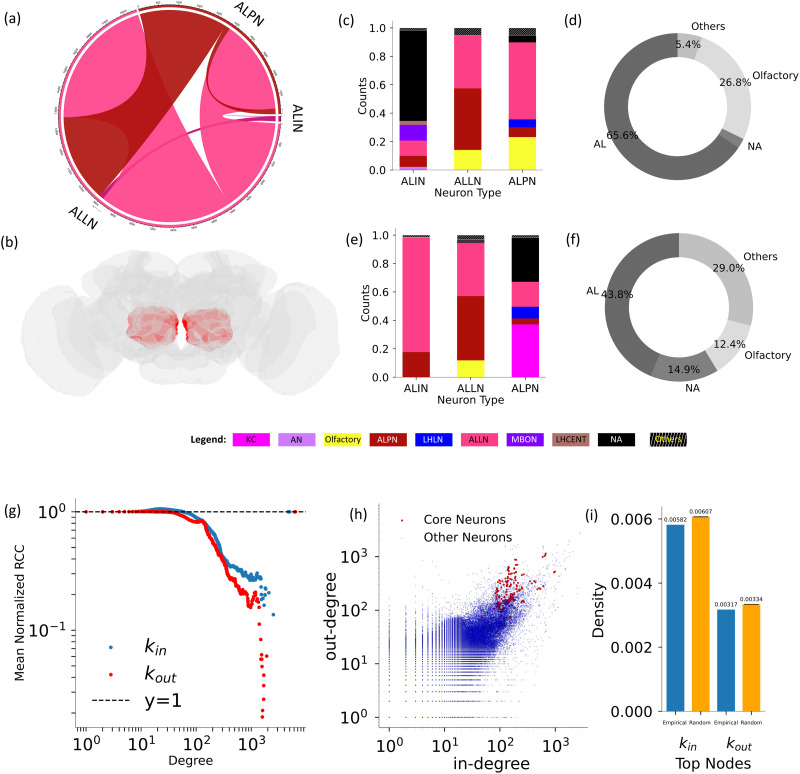
Analysis of the core of the adult brain connectome. The chord diagram in (A) illustrates the neuron types present in the core and the connections among them. Figure (B) displays the adult *Drosophila* brain, with the ALs drawn in red. Figures (C) and (E) display the type composition of predecessors and successors of the different types of core neurons, respectively. Pie charts in (D) and (F) summarize the type composition of predecessors and successors of all core neurons. Figure (G) plots the mean normalized *RCC* versus degree. Figure (H) illustrates the in-degree versus out-degree for all neurons, highlighting the values for the core neurons. Additionally, Figure (I) depicts the density of the top 4.9% high in-degree neurons, top 8.5% out-degree neurons, and the corresponding degree-preserved randomized networks.

We find that the MBNs that formed the core in the larval brain are trimmed off in the early steps (Figure 7) of the D-core decomposition process. Out of the 198 neurons that form the core, 122 are local interneurons, 74 are PNs, and two are antennal lobe input (ALI) neurons (Figure 4A). All these neurons belong to the AL, which serves as the primary center for olfaction in the fly brain. The fly’s AL is analogous to vertebrates’ olfactory bulb. Here, olfactory information from the antennae and maxillary palp reaches the antennal lobe local interneurons (ALLNs) and antennal lobe projection neurons (ALPNs) (Vosshall & Stocker, 2007). After processing in the AL, the information is relayed to the neurons in the MB and lateral horn (LH) (Vosshall & Stocker, 2007). A little is known about ALI neurons; they are labeled as AL-MBDL1 neurons in the hemibrain connectome dataset (Scheffer et al., 2020) and are thought to send feedback signals to the AL. Additionally, they are likely influenced by the activity of MB and LH neurons, as they exhibit arborizations in the ring neuropil, which is connected to these two regions (Tanaka, Endo, & Ito, 2012). The exclusive presence of ALNs in the frontier D-cores could be because of the pivotal role of olfaction in the ecology of an adult fruit fly—locating food sources, finding oviposition sites, avoiding predators, and selecting mates. We also studied the core of the hemibrain connectome dataset (Scheffer et al., 2020) and found that core neurons are mostly the ALNs (Supporting Information Figure S3). Like in the case of larval connectome, we also perform k-core decomposition analyses, which show similar results to D-core analysis as AL neurons are present in the central core (Supporting Information Figure S6), all of which form a subset of the frontier D-core neurons.

Furthermore, to understand the functional role of the core neurons, we investigate the neuronal types that interact with them. Excluding unassigned neurons, most of the in-connections are from other ALLNs and ALPNs for all three types of core neurons (Figure 4C). Thus, all three types show homogeneity in their in-connections, as their predecessors belong to the same neuropil, the AL. We observe that the interspecific connections primarily involve LH local neurons (LHLNs) and olfactory neurons, with a smaller proportion connecting to MBONs.

The pie chart in Figure 4D illustrates a higher contribution from hygrosensory and thermosensory neurons among the interspecific connections. Notably, in the pie chart, the olfactory type is shown separately because it comprises olfactory receptor neurons (ORNs), which are closely associated with the AL. The ORNs send signals to the ALLNs and ALPNs after their odorant receptors bind to odor molecules (Schlegel et al., 2021; Wilson, 2013). Regarding the out-connections of the core neurons, we see a similar dominance of ALLNs and ALPNs, albeit a little diminished because of the significant presence of the KCs as out-neighbors of the ALPNs (Figure 4E). The pie chart in Figure 4F also confirms this. The KC neurons, being part of the MB, are involved in the olfactory associative learning (Li et al., 2020; Lin, Lai, Chin, Chen, & Chiang, 2007), and therefore, the projection of the ALNs to these cells is essential, as reflected in our findings. After the KCs, we find that some of the out-connections of the core neurons are to the LHLN neurons, as is also observed in the in-connections. The first-order connections of core ALNs to LHLNs are expected, as the LH is found to be the higher order processing center of olfactory information in the fruit fly brain (Schultzhaus, Saleem, Iftikhar, & Carney, 2017).

Additionally, we observe that the core of the adult brain comprises the top 4.0% and 8.5% of high in- and out-degree nodes, respectively. The in- and out-degree of the core nodes are highlighted in Figure 4H. It is important to note that these core nodes do not constitute an RC, as the normalized *RCC* takes a value lower than 1 for higher degree values (Figure 4G). However, we also find that the normalized *RCC* exceeds 1 at the intermediate values of the degree, as also shown in the article (Lin et al., 2024) where Dorkenwald et al. (2023) considered that RC neurons were defined as all neurons with degrees above a specified intermediate value where the normalized *RCC* rises above 1.

Using this criterion, we identified a substantial RC comprising 26,662 neurons. However, it is essential to note that, according to the definition of an RC, simply having a high degree does not necessarily qualify nodes as members. Here, the indication of the normalized *RCC* having values lower than 1 indicates the absence of this phenomenon for the high-degree nodes of which the core is part. This is further supported by the lower density of connections among the neurons in the top 4.9% and 8.5% of high in- and out-degree nodes, respectively, compared with their degree-preserved random surrogates (Figure 4I).

### Type Compositions of “Neighbors-of-Neighbors” of Frontier D-Cores’ Neurons

**Figure 5.**
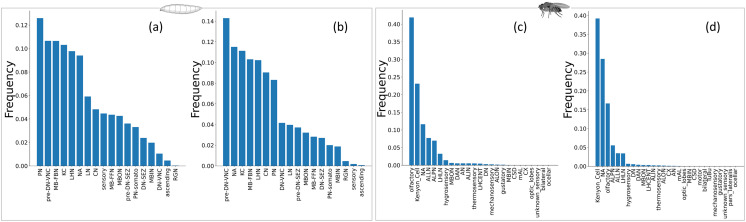
Normalized frequency of different cell types in second-order neighbors of the core. Panels (A) and (B) depict the normalized frequency of predecessors and successors of immediate neighbors of the larval brain core, respectively. Likewise, panels (C) and (D) illustrate the normalized frequency of predecessors and successors of the immediate neighbors of the adult brain core, respectively.

The study, thus far, has demonstrated that both larval and adult core neurons primarily exhibit a similar pattern, characterized by a high proportion of intraspecific connections. Building on these findings, we now aim to explore further by comparing the type diversity of their second-order connections, explicitly examining their neighbors’ neighbors to gain deeper insights into the network’s structure. We find that 25% of the second-order neighbors of the larval core are MB neurons, while 10% of the second-order neighbors of the adult core are AL neurons. At this level, the cell type composition differs significantly between the larval and adult brains. The larval connectome exhibits greater type diversity than the adult connectome among both predecessors and successors of immediate neighbors of the core (Figure 5).

To further quantify this diversity, we calculate both Shannon entropy and Simpson’s index (see the Methods section) to measure the diversity in distribution by considering both type richness and abundance. As shown in Table 2, the higher values of both indices for the larval connectome indicate greater diversity regarding its second-order neighbors.

**Table 2.** Measuring the diversity of the second-order connections

Network	Connection type	Simpson index	Shannon index
Adult	Predecessors of predecessors	0.16	0.13
	Successors of successors	0.13	0.11
Larval	Predecessors of predecessors	0.67	0.22
	Successors of successors	0.62	0.21

The larval connectome exhibits greater diversity in second-order connections compared with adult connectomes, as evidenced by higher normalized Simpson and Shannon indices.

The adult brain shows high second-order associations with olfactory neurons and KCs. In contrast, the cell composition is more diverse for the larval brain. The second-order associations of the core of the larval brain are primarily to the pre-descending ventral nerve cord (VNC) neurons, PNs, MB FBNs, MBONs, LH neurons (LNs), and CNs. The pre-descending VNC neurons are vital in integrating and transmitting nerve signals, coordinating sensory input and motor output, and controlling muscle activities in the periphery (Venkatasubramanian & Mann, 2019). PNs transmit diverse sensory modalities, including olfactory, mechanical, thermal, and gustatory information (Winding et al., 2023). MB FBNs provide feedback from MBONs to help integrate aversive and appetitive memory apparatus (Eschbach et al., 2020). LHNs (lateral horn neurons) are involved in coding odor valence and contributing to innate odor responses (Eschbach et al., 2021). CNs integrate inputs from both the MB, representing learned values, and the LH, representing innate values (Eschbach et al., 2021; Winding et al., 2023). This disparity reflects the broader functional roles played by MBNs in the larval stage compared with the more focused role of the AL in processing chemosensory inputs in the adult stage.

### Topological Roles of the Frontier D-Cores’ Neurons

To further investigate the role of core neurons in the larval and adult brain networks, we examine the impact of their removal on the average clustering coefficient of the entire graph. This is achieved by conducting targeted attacks on both brain networks, observing clustering changes, and global efficiency. In a targeted attack, all frontier neurons are eliminated simultaneously.

**Figure 6.**
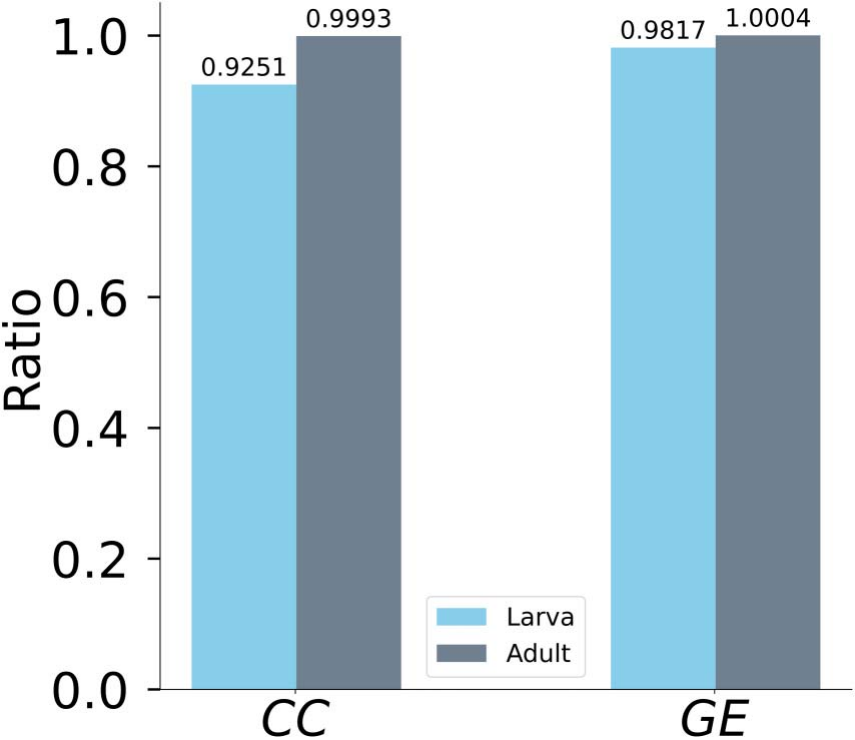
Topological role of the frontier D-cores’ neurons in larval and adult brain. Plots the ratio of the average clustering as well as the ratio of the global efficiency of the brain networks after and before the removal of the D-core neurons.

The bar plots in Figure 6 compare the ratio between the clustering and global efficiency of the network after and before the removal of the D-core neurons in the larval and adult brains, respectively. Figure 6 shows a significant difference between the effect of targeted attacks on the clustering coefficient and the efficiency of the larval brain. However, no such difference is observed in the adult network. In the adult connectome, efficiency appears unaffected, presumably due to the substantial intraconnectivity observed among the ALNs, as illustrated in the type connections heatmap (Figure 8). This suggests that during development, the increase in cell numbers and the formation of dense connections within similar cell types may contribute to resilience against the failure of critical nodes.

### Non-Core MBNs in the D-Cores of the Larval and Adult Brain

**Figure 7.**
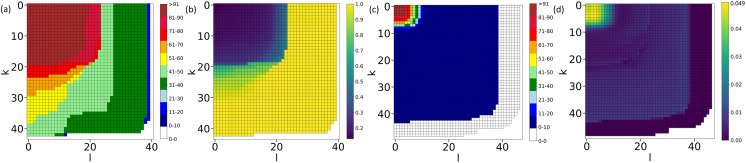
Count of the MBNs in the D-cores of the larval and the adult fruit fly brain. (A) and (C) plot the percentage of the MBNs in the D-core matrix of larval and adult brains, respectively. (B) and (D) plot the ratio of the number of MBNs in a core and the total number of neurons in a D-core for the larval and adult brain, respectively. These plots indicate the dominance of the MBNs in the inner D-cores of the larval brain, whereas the absence of the same in the inner cores of the adult brain.

The observation that MBNs form the core in the larval brain, while not all MBNs are included in the core, raises the question of the positioning of the non-core MBNs within the larval brain. On the other hand, the absence of the MBNs in the adult brain core demands knowledge of the position of these cell types in the core decomposition process. Therefore, we investigate how the MBN population is affected by decomposition in the larval and adult brain networks (Figure 7). In the larval brain, there are a total of 384 MBNs, and this number decreases during decomposition (Figure 7A). This decline is expected as the total number of neurons also decreases. Importantly, all MBNs are part of the inner D-core, and their population is maintained up to the frontier D-cores. The ratio of the MBNs in a D-core to the total number of neurons in that D-core further shows the dominance of the MBNs in the inner D-core of the larval brain (Figure 7A).

In contrast, for the adult brain, which has 5,278 MBNs, the frontier D-cores do not have any of these (Figure 7B). Most MBNs in the adult brain are lost early in the decomposition process. This indicates that the MBNs in the adult brain are mostly connected to low-degree neurons. The D-cores with k ≥ 23 or l ≥ 24 have only two MB type neurons, and for k > 43, none of the D-cores have any MBNs (Figure 7C). Additionally, the k-core analysis (Supporting Information Figure S6) confirms that MBNs are present in the inner cores of the larval brain. At the same time, they exist at the periphery of the adult fly brain.

### Networks of Type-Wise Connections Corroborate D-Core Results

**Figure 8.**
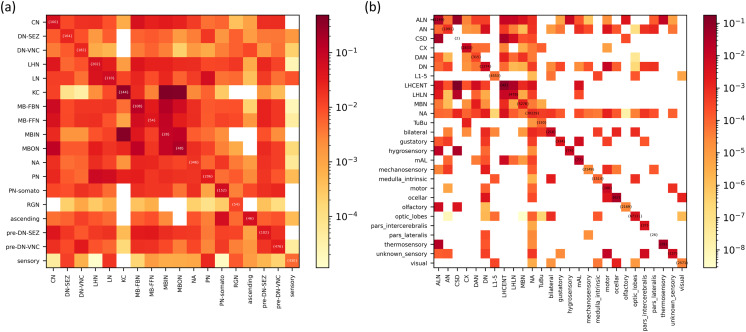
Analysis of the network of type-wise connections. (A) and (D) plot the heatmap of connections between different neuron types of the network normalized by the total possible connections for larval and adult connectome, respectively. The color bar shows the probability of the connection between different cell types. The color intensity represents the logarithmically scaled connection counts, and LogNorm is applied to better emphasize differences across a wide range of values. Diagonal entries show cell counts per neuron type.

We identify two distinct neuron types within the core structures of the larval and adult network, indicating a shift in the cell type composition between the larval and adult brain connectomes. To further elucidate the type-specific behavior within these networks, we construct a network based on connections between different neuron types (Figure 8). As detailed in the Methods section, this network is modeled such that each node represents a distinct neuron type, with edges representing the normalized connections between any two types based on the total possible connections. Normalization accounts for differences in the counts of various cell types. The cell type network is well connected in the larval phase, whereas the adult brain exhibits reduced connectivity among different neuron types, indicating an increased hierarchy level. This further supports the formation of specialized, independent substructures within the brain network during development.

The MBNs exhibit more selective interactions in the adult brain, receiving inputs from ALN, AN (Ascending Neuron), CSD (Contralaterally Innervating Serotonin-Immunoreactive Deutocerebral Neuron), CX (Central Complex Neuron), DAN (Dopaminergic Neuron), DN (Descending Neuron), LHCENT (Lateral Horn Centrifugal Neuron), LHLN (Lateral Horn Local Neuron), and other MBNs. Their outputs are directed toward ALN, AN, CX, DAN, DN, LHCENT, LHLN, other MBNs, and TuBu (Tubercle Bulb) (Figure 8). At the same time, the ALNs are characterized by extensive connectivity, engaging with numerous neuron types, in addition to those mentioned for the MBNs, such as gustatory, mAL (Mediodorsal Antennal Lobe), mechanosensory, motor, olfactory, paralateral, and thermosensory neurons. None of these neuronal cell types were present in the data for the larval phase. This also suggests that the shift in the core from MBNs to ALNs may result from limited connectivity between MBNs and the new cell types that emerge in the adult brain to support phase-specific functions.

Furthermore, to show a change in the centrality of MBNs in the adult brain compared with the larval brain, we compute

$${\langle btw\rangle }_{\text{MBNs}}=\frac{\langle {btw}_{\text{MBNs}}\rangle }{\langle {btw}_{\text{whole}}\rangle },$$ (1)

where 〈*btw*_MBNs_〉 is the average betweenness centrality of the MBNs and 〈*btw*_*whole*_〉 is the average betweenness centrality of the whole network. We find that 〈*btw*〉_MBNs_ for the larval brain is 1.33, showing that the average betweenness centrality of the MBNs is much higher than that of the whole network (Table 1). Meanwhile, for the adult brain, this ratio is 0.68, indicating that the MBNs have a much lower betweenness centrality value than the average of the whole network. This suggests that MBNs, which are highly central in the larval brain, become less central in the adult brain. Moreover, the above ratio 〈*btw*〉_ALNs_ for the ALNs in the adult brain is 2.7, way higher than the 0.68 value observed for the MBNs, assuring a shift in the centrality in the adult phase from the MBNs to the ALNs.

To further identify the core in the cell type network, we implemented an s-core decomposition analysis (Eidsaa & Almaas, 2013) (see the Methods section). In the larval network, KCs, MBINs, and MBONs are identified as forming the in-degree s-core, while KCs and MBONs comprise the out-degree s-core. This supports the results obtained from the D-core analysis of the entire neuronal network.

After grouping all AL and MB neurons in adult networks, we find that AL neurons, LHLNs, LHCENTs, and CSD neurons form the s-core for both in-degree and out-degree metrics. LHCENT neurons play a crucial role in feedback mechanisms to the LH and the MB calyx (Bates, Schlegel, et al., 2020), originating from areas targeted by the LH, including the superior lateral protocerebrum, superior intermediate protocerebrum, and superior medial protocerebrum, which represent key centers for third-order olfactory processing (Das Chakraborty & Sachse, 2021). CSD neurons, known for serotonin production in *Drosophila*, modulate olfactory networks through specific inputs from ORNs and PNs associated with distinct glomeruli, in addition to receiving widespread inputs from local interneurons (LNs). The output from CSD neurons differs across glomeruli and remains consistent within individuals (Coates et al., 2017). Notably, within the s-cores, we observe that the connectivity profile of AL neurons is much broader than that of the other neuron types: CSD, LHLN, and LHCENT neurons receive and send signals to a limited number of neuron types. This suggests that ALNs are indeed the neural core of the adult brain network.

## DISCUSSION

We compare the larval and adult fruit fly neuronal brain networks to analyze the developmental changes occurring during the growth of the fly brain throughout metamorphosis. Brain rewiring during fly development involves metamorphosis characterized by neuronal death, brain reorganization, and the development of adult-specific neurons. Therefore, larval and adult life stages are expected to differ significantly, each linked to stage-related functions and environmental stimuli. The study aims to pinpoint the significant changes during the developmental process.

We find significant alterations in the mesoscopic organization of brain connectivity as *D. melanogaster* evolves from the larval to the adult phase. However, we also notice certain similarities between the two brain networks; both exhibit sparsity and a modular organization likely preserved through evolutionary processes, which were previously reported (Gerhard, Andrade, Fetter, Cardona, & Schneider-Mizell, 2017).

We find that the adult brain is more modular than the larval brain, reflecting an enhancement of specialized brain compartments with distinct functions. This enhancement is also reflected in the reduction in density. Overall, this suggests that the adult brain has evolved to feature specialized structural compartments, which likely serve as functional epicenters. This organized integration has been previously reported and demonstrates how brains can systematically process sensory information into structured feature maps (Klapoetke et al., 2022). These maps form diverse pathways that are essential links between vision and behavioral controls, highlighting the brain’s ability to coordinate complex functions efficiently.

Moreover, the adult degree distribution shows a stronger conformity to a power-law, with its out-degree distribution falling within the scale-free domain and the in-degree distribution narrowly failing to meet the threshold. In contrast, the larval degree distribution fits the Weibull distribution, with its growth parameter (*β*) greater than 1. This difference between the two stages indicates that as the network grows, it adapts to have the scale-free property to gain robustness against failures while minimizing the cost associated by decreasing the density. A recent study of the brain network of *C. elegans* across its development found that degree distributions consistently did not follow a power-law model across all stages (Zhao et al., 2022). This observation underscores the intricacies inherent in the developmental process of *Drosophila* compared with *C. elegans*, a distinction that reflects the fact that *Drosophila* undergoes complete metamorphosis.

Further analysis of the core of larval and adult brain networks using D-core decomposition, which is analogous to the k-core of undirected networks, reveals that the larval core comprises MBNs. In contrast, the adult core consists of ALNs. These differences in core constituents for the adult and larval connectome may reflect their disparate lifestyles. The exclusive participation of MB neurons in the larval brain core indicates that the MB neurons have a central role in its functioning. This conclusion is supported by recent researches (Eichler et al., 2017; Technau, 2009; Vosshall & Stocker, 2007), indicating that the MB not only serves as the hub for olfactory learning but also integrates multiple sensory inputs for learning and memory. This is because KCs receive thermal, gustatory, and visual input through nonolfactory PNs, and these multisensory KCs and MBINs synapse onto MBONs.

In addition, the analysis of the neighbors of the core provides an understanding of the neuronal pathways and the role of different types of neuronal cells in forming a complex yet robust network architecture. The core’s cell type composition is retained in its immediate neighbors in both the larval and the adult brain. Specifically, the core neurons and their immediate neighbors are located within a single neuropil: the MB in the larval brain and the AL in the adult brain. Moreover, the larval network exhibits a strong RC organization in both the in-degree and out-degree distributions. The RC contains a majority of neuron types within its core, demonstrating significant interconnectivity among various cell types in the larval brain. This suggests that, during the initial phases of development, distinct brain regions exhibit a high degree of connectivity.

The core being part of the RC is more connected than the RC as a whole, indicating the existence of a “rich club within the rich club.” This suggests the hierarchical organization of connectivity, which may have implications for functional integration and information processing. This observation also indicates that the core of the larval connectome has a global influence on the network, which is supported by a significant decrease in the average clustering coefficient and global efficiency of the larval connectome when we remove the core nodes. On the other hand, the core of the adult brain does not form the RC and, therefore, exerts a peripheral influence, evidenced by the negligible effect on its average clustering coefficient and global efficiency after the loss of core nodes. This could be due to the large number of well-connected ALNs in the adult brain compared with the larval brain.

Based on the diversity of the types of second-order neighbors of core neurons, it can be inferred that the larval core is more generalized and connects with a diverse range of cell types. In contrast, the adult core has localized connections, extending mostly to the olfactory neurons. At the same time, the cell type connection matrices show that the MBNs in the adult brain are not connected to most of the neuronal cell types that were not present in the larval dataset. This aligns with lower betweenness centrality values of the MBNs observed in the adult brain than in the larval brain, further confirming the shift in the centrality of MBNs.

The larval connectome’s globally influential core may result from the predominance of MBNs, which perform diverse roles in the larval phase of the fly. After metamorphosis, observation of a shift in the core and the reduction in the centrality of the MBNs may be related to the inclusion of the new cell types and the biases in their connections due to potential shifts in the functional or behavioral demands on the brain network during adulthood. However, the exact nature of these changes remains to be fully understood.

Furthermore, although all core neurons in the adult stage belong to a single neuropil, and the same holds for the larval stage, not all neurons within these neuropils are part of the core. This suggests that, at the network scale, hierarchy exists not only among different cell types but also within similar cell types.

*D. melanogaster* undergoes complete metamorphosis, comprising four stages: egg, larva, pupa, and adult. The evolutionary theory posits that metamorphosis evolved to mitigate resource competition between young and adults, enabling larval and adult forms to occupy distinct ecological niches (Ten Brink, de Roos, & Dieckmann, 2019). This ecological divergence likely contributes to the extensive brain rewiring observed during development. The significant distinction between the topology of the brain network of the two different phases of the *D. melanogaster* is also in agreement with other findings that despite some of the neuronal cells surviving the metamorphosis, their connection completely changes, and there is no evidence of the larval memory persisting into adulthood (Truman et al., 2023). The comprehensive comparison of adult and larval connectomes offers valuable insights into the complexity of developing brain networks in *D. melanogaster*. It serves as a foundational framework for understanding the mechanisms underlying brain network development and provides a platform for future investigations.

Furthermore, we acknowledge certain limitations in comparing the two datasets. For example, distinguishing between various synapse types (e.g., excitatory vs. inhibitory, axo-dendritic, dendro-dendritic, axo-axonal) could provide additional insights into the network’s connectivity patterns. However, the excitatory/inhibitory information is not available for the larval brain; at the same time, axo-dendritic, dendro-dendritic, and axo-axonal connections are not available for the adult brain dataset. Consequently, a detailed comparative analysis based on synapse type is currently not feasible. Additionally, since these datasets represent individual females, a generalized understanding could only be obtained from future investigations into more connectomes. Given these limitations, our study adopts a broader approach, examining overall connectivity to understand how the topology differs across developmental stages in *Drosophila*’s brain. Our primary focus is on establishing fundamental insights into the overarching structural connectivity, thereby highlighting key developmental differences in brain organization between the larval and adult stages.

## METHODS

### Data Collection and Analysis of Network Structural Properties

The larval and adult *Drosophila* brain network datasets are collected from Winding et al. (2023) and Matsliah et al. (Matsliah et al., 2023), respectively. The nodes in these networks represent different neurons, and the edges are the synaptic connections between them. We extract the respective tables representing neuronal connections from both datasets and construct binary network representations for the larval and adult stages using the Python package NetworkX (Hagberg, Swart, & Schult, 2008).

The adjacency matrix of each network is denoted as *A*, where each element *A*_*ij*_ = 1 indicates the presence of a synapse from Neuron *i* to Neuron *j*. Binarizing the network helps emphasize essential pathways that contribute to the brain’s small-world architecture. By focusing on the degree rather than the weighted degree, we aimed to prevent the underrepresentation of weak connections, which might play a pivotal role in linking specialized brain regions, aligning with the “strength of weak ties” theory observed in social networks (Granovetter, 1973) and in the functional brain networks (Gallos, Makse, & Sigman, 2012). Both the datasets are directed and, hence, *A*_*ij*_ ≠ *A*_*ji*_. One of the structural measures of a network is the node degree (*k*_*i*_ = ∑_*j*_ *A*_*ij*_), which quantifies the number of edges linked to a given node. The density measures how well connected the nodes are within the network and is defined as the ratio of the number of actual edges present in the network to the total number of possible edges. For a directed network, it is given as *D* = $\frac{E}{N\left(N-1\right)}$, where *E* is the total number of edges and *N* is the total number of nodes present in the network. Furthermore, the directed clustering for a node *i* is defined as (Fagiolo, 2007): *C*_*i*_ = $\frac{L_{i}}{k_{i}\left(k_{i}-1\right)}$, where *L*_*i*_ is the number of directed links between the *k*_*i*_ neighbors of node *i*, and *k*_*i*_ = *k*_*in*_ + *k*_*out*_ is the sum of the in- and out-degree of node *i*. All these structural properties were calculated with the help of the Python library NetworkX (Hagberg et al., 2008). Furthermore, the global efficiency is the average of the efficiencies of all the pairs of nodes in a network, given as follows: *E*_*global*_ = $\frac{1}{N\left(N-1\right)}\sum_{i\neq j\epsilon V}\frac{1}{d_{ij}}$. The Python package igraph is utilized for efficiency calculations.

Furthermore, a good measure of the influence of a node over the entire network is its betweenness centrality. We use betweenness centrality to compare the impact of the core neurons between the larval and adult brain networks. For a node *v*, it is defined as: *c*_*B*_(*v*) = ∑_*s*≠*v*≠*t*_ $\frac{\sigma_{st}\left(v\right)}{\sigma_{st}}$, where ∑ *σ*_*st*_ is the number of the shortest path between all the pair of nodes *s* and *t* and *σ*_*st*_(*v*) is number of shortest paths that pass through the node *v*. Please note that all betweenness centrality values have been calculated for the largest strongly connected component.

### Community Detection Using the Louvain Algorithm

For the larval connectome, consensus community detection is performed by running 100 instances of the algorithm using 100 different random seed values at the default resolution. Then, we count the number of times any two nodes feature in the same community and construct a co-occurrence matrix. Lastly, we run a community detection algorithm on this matrix to obtain the final list of communities. In the case of the adult brain network, the computational complexity arising from the vast size of the co-occurrence matrix and the iterative process of updating the matrix with co-occurrences for each pair of nodes rendered the execution of a consensus community detection as detailed above infeasible. Consequently, we opted to employ a single instance of community detection at the default resolution. The partitions obtained from the community detection of both brain networks are then used to obtain the value of modularity. Modularity for a directed graph is defined as follows (Clauset, Newman, & Moore, 2004): *Q* = $\sum_{c=1}^{n}\left[\frac{L_{c}}{m}-\gamma {\left(\frac{k_{c}^{\text{in}}k_{c}^{\text{out}}}{2m}\right)}^{2}\right]$, where the sum is taken over all the *n* communities, *L*_*c*_ is the number of intracommunity links for the community *c, m* is the total number of edges in the network, *γ* is the resolution parameter, and $k_{c}^{\text{in}}$ and $k_{c}^{\text{out}}$ are the sum of in-degrees and out-degrees respectively in community *c*.

### Degree Distribution and Fitting

For fitting the degree distributions to the Weibull and power-law model, we use the Python power-law package created by Alstott, Bullmore, and Plenz (2014), which utilizes the statistical techniques laid down by Clauset et al. (2009). Simply put, the optimum minimum value of data (*x*_*min*_) is selected for which the Kolmogorov–Smirnov distance is the least. Then, the value of the exponent is calculated using MLE. MLE gives a more accurate estimation of the power-law exponent than linear regression on doubly logarithmic axes because the latter has issues with hard-to-estimate errors, unreliable statistical measures (*r*^2^ values), and lack of normalization (Clauset et al., 2009).

### D-Core Decomposition Method

The D-core decomposition is carried out using the algorithm laid out in the paper by Giatsidis et al. (2013), applied to both the larval and adult connectomes. Given a graph *G*, we can obtain a unique D-core(*k*, *l*) (*DC*_*k*,*l*_), which is a maximal subgraph that contains nodes of in-degree ≥ *k* and out-degree ≥ *l*. D-core is obtained for all pairs of *k* and *l* values such that for each pair, k ≤ *k*_*max*_ + 1 and l ≤ *l*_*max*_ + 1. Subsequently, we define D-core matrix of the network *G* as *A*_*G*_(*k*, *l*) = (*dc*_*k*,*l*_)_*k*,*lϵN*_, where *dc*_*k*,*l*_ is the size of the D-core(k, l) (see Figure 2A). We implement the algorithm mentioned in the Supporting Information for all pairs of *k* and *l* values such that for each pair, k ≤ *k*_*max*_ + 1 and l ≤ *l*_*max*_ + 1. The D-core matrix is drawn until *l*_*max*_ + 1 and *k*_*max*_ + 1 on the *x*- and *y*-axis, respectively, where *l*_*max*_ is the value of *l* such that *DC*_0,*lmax*_ is the last non-empty graph along the *x*-axis and, similarly, *k*_*max*_ is the value of *k* such that *DC*_*kmax*, 0_ is the last non-empty graph along the *y*-axis (see Figure 2A). The last non-empty D-cores, w.r.t. increase in *k* and *l* increase, are highlighted with white colored oblique lines in the Figures 2B and 2C. These are termed as the frontier D-cores.

### RC Analysis

We calculate the in-degree and out-degree *RCC* defined as follows (Van Den Heuvel et al., 2013):

$$RCC\left(k_{\text{in}/\text{out}}\right)=\frac{M_{>k_{\text{in}/\text{out}}}}{N_{>k_{\text{in}/\text{out}}}\left(N_{>k_{\text{in}/\text{out}}}-1\right)},$$

where *N*_>*k*_*in*/*out*__ is the number of nodes with in-/out-degree > *k*_*in*/*out*_ and *M*_>*k*_*in*/*out*__ is the number of edges between them. As the high-degree nodes naturally have more probability of connecting with nodes of any type of degree, including the high-degree one, the normalization of the above by the corresponding degree sequence preserved randomized networks provides more accurate assessment of RC organization (Colizza, Flammini, Serrano, & Vespignani, 2006). Therefore, we normalized the *RCC* calculated for the brain network with the *RCC* for the corresponding 100 degree preserved random networks.

### Degree Preserving Random Networks

To investigate if a graph truly shows RC organization, we normalize its *RCC* with that of degree-preserved random graphs. Such a mode of randomization only changes the arrangement of the edges, keeping the degrees of the nodes unchanged. This is carried out by implementing the directed_edge_swap method in the NetworkX (Hagberg et al., 2008) package. For both networks, 100 degree-preserved random networks are created.

### Targeted Attack

Attack experiments are carried out to discern the effect of the loss of core neurons in both brain networks. Targeted attacks involve removing the entire set of core neurons at the same time and then measuring the parameters. For calculating the directed global efficiency, we consider the largest and strongest component, whereas for the average directed clustering coefficient, we consider the entire graph.

### Diversity Indices

To assess the diversity in the neuron types of second-order neighbors of the core neurons, we implement two diversity indices: Simpson’s index (*S* = $\frac{n}{\sum_{i}^{n}p_{i}^{2}}$) and Shannon index/entropy (*H* = −$\sum_{i}^{n}\frac{p_{i}\ln p_{i}}{\ln n}$), where *p*_*i*_ = *N*_*i*_/*N* refers to the probability of a cell type, *n* is the total number of cell types studied, *N*_*i*_ is the number of *i*th cell type, and *N* is the total number of neurons studied. These measures were normalized to account for the different sizes. In the case of the Shannon index, this is done by taking the logarithm of the set of neurons being considered. The Shannon entropy is further utilized to assess how the diversity of D-cores changed during the process of decomposition.

### Construction of Type-Wise Connectivity Networks

For both brain networks, we create an adjacency matrix showing connections within and between different types of neurons. The values are normalized by the total number of possible connections, as demonstrated by the following formula:

$$P_{ij}=\left\{\begin{array}{ll} \frac{E_{ij}}{N_{i}\times N_{j}}, & \text{if}\;i\neq j\\ \frac{E_{ii}}{N_{i}\left(N_{i}-1\right)}, & \text{if}\;i=j \end{array}\right.$$

where *P*_*ij*_ denotes the probability of connection from *i*th neuron type to the *j*th neuron type, *E*_*ij*_ is the number of connections from *i*th neuron type to the *j*th neuron type in the network, and *N*_*i*_/*N*_*j*_ represent the count of neurons of type *i* and *j*, respectively. This matrix is used as the adjacency matrix for further network analysis.

### k-Core Decomposition

k-core decomposition prunes a graph based on the nodes’ unweighted degrees iteratively. Given a graph *G*, the k-core is the maximal subgraph in which every node has a degree of at least k. The k-core decomposition involves iteratively pruning the graph by removing nodes whose degree is less than k for increasing values of k until no more such nodes remain. This decomposition results in a series of nested subgraphs, or k-cores. We performed the k-core decomposition on the total degree of the larval and adult fly’s brain networks.

### s-Core Decomposition

s-core is the extension of the k-core method for the weighted networks. Given a weighted graph *G*, the s-core is the maximal subgraph in which every node has a degree of at least s. The s-core decomposition involves iteratively pruning the graph by removing nodes whose degree is less than s for increasing values of s until no more such nodes remain. This decomposition results in a series of nested subgraphs, or s-cores.

### Packages Used for Analysis

We carry out the analysis using the following Python packages: igraphs (Csardi & Nepusz, 2006), NetworkX (Hagberg et al., 2008), numpy (Harris et al., 2020), pandas (McKinney, 2010), power-law (Alstott et al., 2014), and scipy (Virtanen et al., 2020), whereas plotting is done using fafbseg (Dorkenwald et al., 2023; Schlegel et al., 2023), matplotlib (Hunter, 2007), navis (Bates, Manton, et al., 2020), and seaborn (Waskom, 2021).

## ACKNOWLEDGMENTS

A.S. acknowledges the Department of Science and Technology (DST), Government of India, for financial support through grants DST/INSPIRE/04/2021/001893. P.Y. acknowledges UGC for providing financial support under the Junior Research Fellowship scheme and the lab members for their timely assistance and valuable discussions. We also express gratitude to IISER Tirupati for providing the High-Performance Computing facility. Additionally, we are thankful to Prof. Sitabhra Sinha from the Institute of Mathematical Sciences, Chennai, for his insightful comments on this work.

## SUPPORTING INFORMATION

Supporting information for this article is available at https://doi.org/10.1162/NETN.a.26.

## AUTHOR CONTRIBUTIONS

Aradhana Singh: Conceptualization; Formal analysis; Funding acquisition; Investigation; Methodology; Project administration; Software; Supervision; Validation; Visualization; Writing – original draft; Writing – review & editing. Prateek Yadav: Conceptualization; Data curation; Formal analysis; Methodology; Resources; Software; Visualization; Writing – original draft; Writing – review & editing. Pramod Shinde: Formal analysis; Project administration; Software; Supervision; Validation; Visualization; Writing – original draft; Writing – review & editing.

## FUNDING INFORMATION

Aradhana Singh, Department of Science and Technology, Ministry of Science and Technology, India (https://dx.doi.org/10.13039/501100001409), Award ID: DST/INSPIRE/04/2021/001893.

## Supplementary Material



## TECHNICAL TERMS

*Drosophila melanogaster*: A fruit fly species widely used as a genetic and neuroscience model organism due to its short life cycle, well-mapped genome, and tractable nervous system.
Metamorphosis: A biological process involving a marked and relatively sudden transformation in body structure, typically beginning at birth or hatching, driven by cell growth and differentiation.
D-core decomposition: An iterative method that prunes a directed graph to extract maximal subgraphs where all nodes meet minimum in- and out-degree thresholds.
Modularity: A measure of how well a network divides into clusters (modules) with denser connections within than between them.
Scale-free network: A network whose degree distribution follows a power-law with the exponent being 2–3 (the scale-free regime), with few highly connected hubs and many sparsely connected nodes.
Mushroom body: An insect brain region primarily involved in learning, memory, and sensory integration.
Antennal lobe: The primary olfactory processing center in insects, analogous to the vertebrate olfactory bulb.
Betweenness centrality: Measures how often a node lies on the shortest paths between other nodes, indicating its role in information flow.
Network’s core: This maximal subnetwork is obtained by iteratively removing the low-degree nodes. It integrates the entire network and, therefore, serves as the core.
Rich club coefficient: A metric quantifying the tendency of high-degree nodes to connect more densely with each other than expected by chance.
Olfaction: The sense of smell in Drosophila involves detecting airborne chemical cues through sensory neurons in the antennae and maxillary palps, which relay signals to the antennal lobe for processing.
