# Supplementary material for: Brain rewiring during development: A comparative analysis of larval and adult Drosophila melanogaster connectomes
Source: Netw Neurosci. 2025 Nov 20;9(4):1299–322. doi: 10.1162/NETN.a.26 (PMC12635837; doi:10.1162/NETN.a.26)
Supplement: Supplementary file 1 [file netn-9-4-1299-s001.pdf]

Brain rewiring during development: A Comparative Analysis of Larval and Adult *Drosophila melanogaster* Connectomes Supplementary Materials

Prateek Yadav[a], Pramod Shinde[b], and Aradhana Singh[c, *]

[a]Department of Biology, Indian Institute of Science Education and Research Tirupati, India
[b]La Jolla Institute for Immunology, La Jolla, CA, USA
[c]Department of Physics, Indian Institute of Science Education and Research Tirupati, India

Degree Distribution: Here we show that both the in and out-degree of the larval well fit poorly with the power law and both the in and the out-degree of the adult brain fit poorly with the Weibull distribution (Figure S1 (a), (b)).

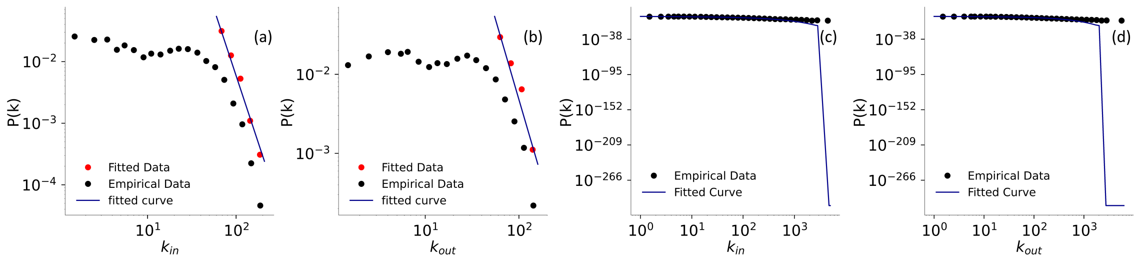

Figure S1: **In and out-degree distribution of the larval (a, b) and the adult (c, d) brain networks**. This shows the poor fitting of the larval in- and out-degree with the power-law and adult brain's in- and out-degree with the Weibull distribution.

Algorithm for the D-core decomposition: D-core decomposition was carried out using the algorithm laid out in the paper by Giatsidis *et al.* [1]. Given a network called G, we can obtain a unique D-core(k, l), $DC_{k,l}$ which is a maximal subgraph that contains nodes of in-degree ≥ k and out-degree ≥ l. We implemented the algorithm in Python as follows:

INPUT: a directed graph G and positive integers k, l
OUTPUT: adjacency matrix corresponding to $DC_{k,l}$, nodes belonging to $DC_{k,l}$
1.1 initialise nodesarray
1.2 initialise adjacency matrix
1.3 recursion = True
2 while recursion == True and matrix is not empty
2.1 nodes to be deleted = nodes with either in-degree ≤ k OR out-degree ≤ l
2.2 remove the nodes to be deleted from the adjacency matrix
2.3 if nodes to be deleted is empty
2.3.1 recursion = False
end
end;

The statistics of the maximal likelihood test performed to fit the data with the power-law distribution are tabulated in Table S1:

		Adult		Larva	
		In-degree	Out-degree	In-degree	Out-degree
γ		3.15	2.96	4.34	4.06
**KS distance**		0.01	0.02	0.05	0.08
x_{min}		113	90	60	55
**Exponential**	LR	8.09	6.33	-3.30	-8.38
	p	6×10^{-6}	2.51×10^{-10}	9.72×10^{-4}	5.13×10^{-17}
**Truncated Power Law**	LR	0.19	-0.17	-4.14	-9.52
	p	0.97	0.82	5.02×10^{-8}	1.78×10^{-8}
**Log normal**	LR	1.73	0.0049	-3.29	-5.59
	p	0.084	0.996	0.0009896	2.26×10^{-8}
**Stretched Exponential**	LR	3.8	2.0638	-3.39	-5.84
	p	1.44×10^{-4}	0.0390	0.000696	5.29×10^{-9}
**Lognormal Positive**	LR	3.59	2.0965	-3.29	-5.59
	p	3.27×10^{-4}	0.0360	0.0009896	2.26×10^{-8}

Table S1: Tests of Power Law behavior of degree distributions in Adult and Larva Connectome. LR stands for loglikelihood ratio. Positive values for the loglikelihood ratio (LR) indicate a preference for the power law model over alternative models when the p-value is less than 0.05. However, if the p-value exceeds 0.05, the sign of LR becomes an unreliable indicator of which model provides the better fit to the data.

Further, we calculated the normalized Shannon entropy for all the D-cores of the Larval and the Adult brain network. The classification of neurons in the adult fly dataset is done using hierarchical annotations while the data for the larval connectome provides primarily the cell-type classification. Therefore, to have more overlap between the cell types of the larval connectome with the adult connectome, we have considered the 'class' annotation of the adult fruitfly dataset. We find that for both brains, the heterogeneity in the cell type increases as we move from the outer to the inner cells and then decreases (Figure S2). This decrement towards the inner cores is more in the larval brain as compared to the adult brain.

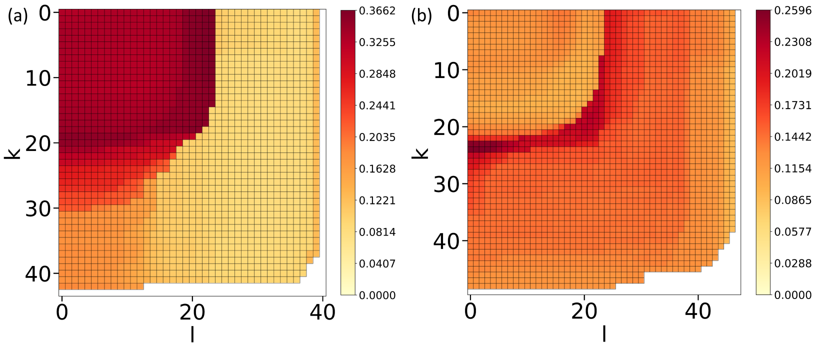

Figure S2: **Heterogeneity of the D-cores as per the cell types** (a) and (b) plot the normalized Shannon entropy for the different D-core of the Larval and the Adult brain networks, respectively.

D-core of the Hemibrain dataset:

We also implement D-core decomposition on the Hemibrain dataset [2], which maps 20 million synapses between 25,000 neurons in the central brain of the 5-day-old female fruit fly. Specifically, we apply the decomposition on the binary network of strong connections, defined as those with 10 or more synapses. In this network, the rate of trimming of the neurons is faster compared to the whole brain data (Figure S3 (a)). However, here too, we find that the ALNs mainly form the core. As plotted in Figure S3 (b), out of the 88 frontier D-cores' (structural core) neurons, there are 60 ALLNs, 26 ALPNs, and 2 Octopaminergic neurons. This is in agreement with the observation for the whole brain dataset, confirming the ALNs are the structural core of the adult fruit fly's brain network.

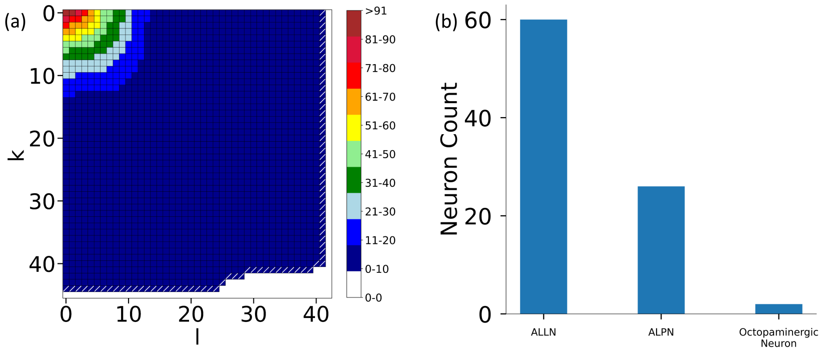

Figure S3: **D-core study of the Hemibrain dataset.** Subplot (a) shows the D-core matrix of the Hemibrain dataset. The color bar plots the percentage of the remaining nodes at the different levels of the D-cores, indicating that the frontier D-cores have $0 - 10\%$ of the neurons. The subplot (b) shows the type composition of the core obtained.

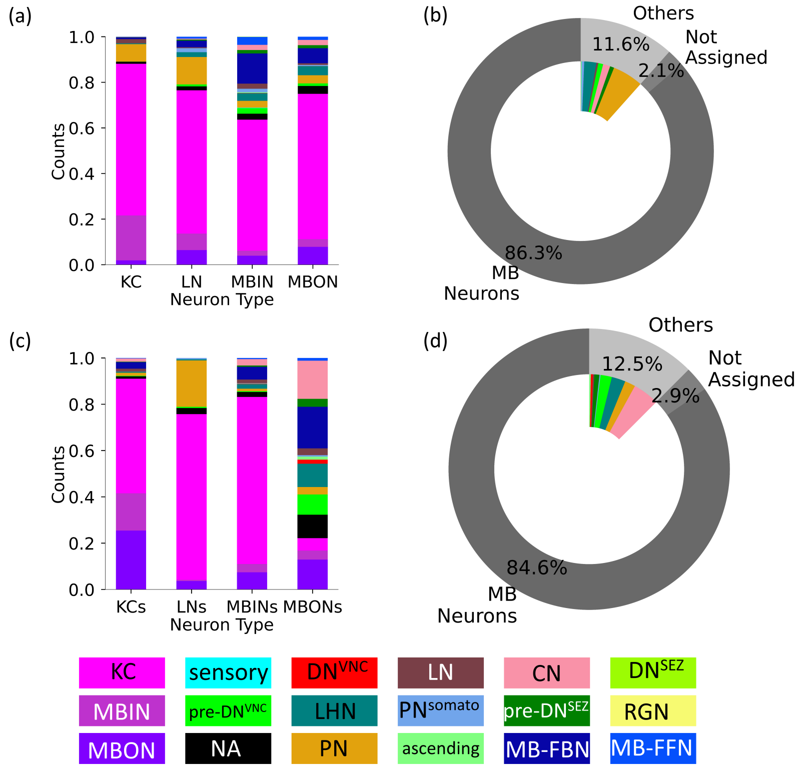

Figure S4: **Analysis of the neighbors of larval core** In (a) and (c), the normalized cell type composition of predecessors and successors of the different types of core neurons in the larva connectome are plotted. Pie charts in (d) and (f) summarize the type composition of predecessors and successors of all core neurons. All the types are shown without applying any threshold.

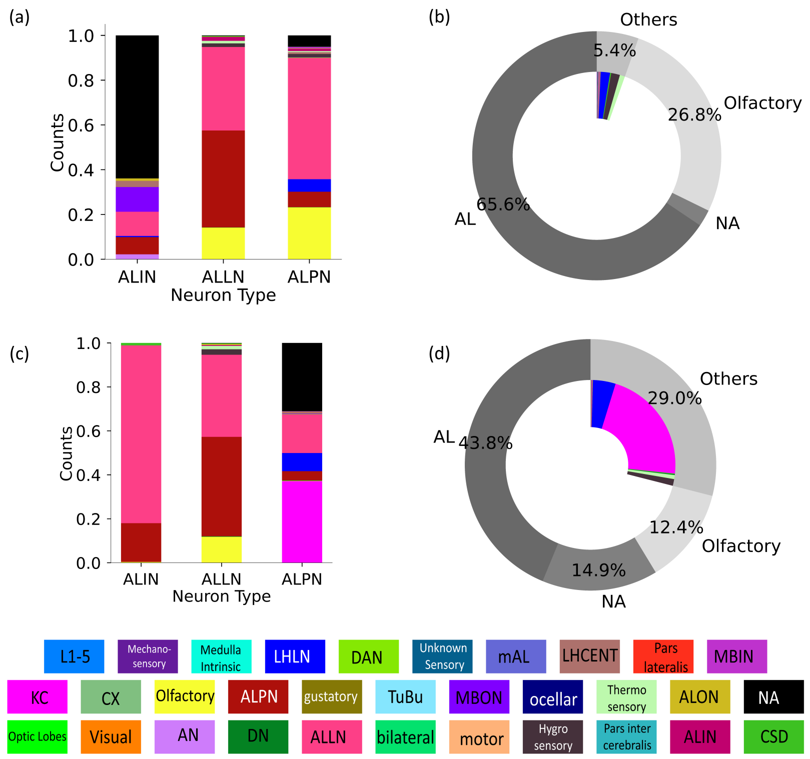

Figure S5: **Analysis of the neighbors of adult core** In (a) and (c), the normalized cell type composition of predecessors and successors of the different types of core neurons in the adult connectome are plotted. Pie charts in (d) and (f) summarize the type composition of predecessors and successors of all core neurons. All the types are shown without applying any threshold.

k-core decomposition confirms the D-core analysis of both the brain networks We observed that the k-core findings support the D-core analysis in both networks: both main k-cores are a subset of the neurons of the frontier D-cores. In the case of the larva, the innermost core contains 134 neurons (107 KC, 9 MBON, 16 MBIN and 2 LN) while in the case of the adult, the innermost core comprises of 121 neurons (94 ALLN and 27 ALPN) (Figure S6).

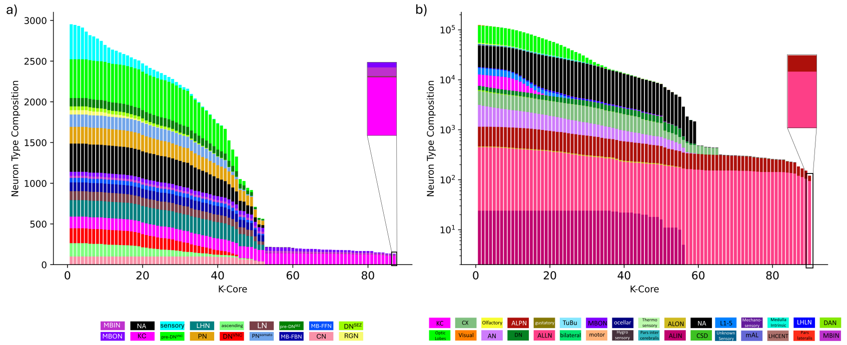

Figure S6: **Neuron Type Composition of k-cores of the larval and the adult brain networks**. The type composition of the different k-shells is plotted for the larval (a) and adult (b) brain network. In the larval brain, the composition of the last shell, the core, is of MBNs, whereas in the adult brain, the core comprises the ALNs. The k-core decomposition is performed considering the total degree of the network.

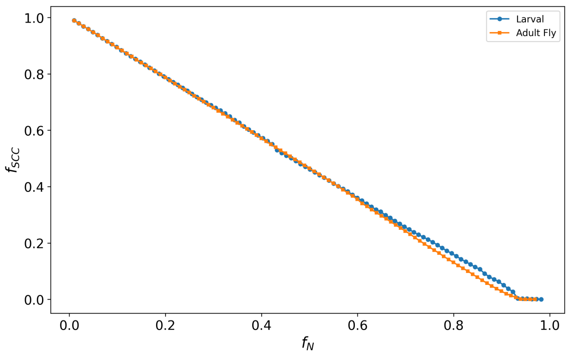

Figure S7: **Robustness of the larval and adult brain.** This figure illustrates the relationship between the fraction of nodes removed and the fraction of the largest strongly connected component (f_{SCC}) in the network for the larval and adult connectomes as nodes are randomly removed. In each step, 1% of the nodes are removed, and the size of the SCC is calculated.

Robustness of the larval and the adult brain to the error propagation: To facilitate a direct comparison of network robustness, we conducted an error percolation analysis that demonstrates both the larva and adult connectomes exhibit similar profiles (Figure S7), despite the adult connectome being significantly sparser. This observation may be due to the power-law characteristics of its degree distributions and the scale-free nature of its out-degree distribution. Scale-free networks have previously been reported to be robust to error propagation [3].

**Acronym**	**Full Form**
MBON	Mushroom Body Output Neuron
MBIN	Mushroom Body Input Neuron
KC	Kenyon Cell
NA	Not Assigned
pre-DN^{VNC}	Predescending Neuron to the Ventral Nerve Cord
PN	Projection Neuron
LHN	Lateral Horn Neuron
DN^{VNC}	Descending Neuron to the Ventral Nerve Cord
DN^{SEZ}	Descending Neuron to the Subesophageal zone
PN^{somato}	Somatosensory Projection Neuron
LN	Local Interneuron
MB-FBN	Mushroom Body Feedback Neuron
pre-DN^{SEZ}	Predescending Neuron to the Subesophageal zone
CN	Mushroom Body-Lateral Horn Convergence Neuron
MB-FFN	Mushroom Body Feedforward Neuron
RGN	Ring Gland Neuron
L1-5	Lamina Monopolar 1 to 5
CX	Central Complex Neuron
AN	Ascending Neuron
DN	Descending Neuron
ALPN	Antennal Lobe Projection Neuron
LHLN	Lateral Horn Local Neuron
ALLN	Antennal Lobe Local Interneuron
DAN	Dopaminergic Neuron
TuBu	Tubercle Bulb Neuron
mAL	mediodorsal Antennal Lobe Neuron
LHCENT	Lateral Horn CENTrifugal Neuron
ALIN	Antennal Lobe Input Neuron
ALON	Antennal Lobe Output Neuron
CSD	Contralaterally innervating Serotonin immunoreactive Deutocerebral interneuron
AL-MBDL1	Antennal Lobe Median BunDLe Neuron
ORN	Olfactory Receptor Neuron

Table S2: Table displaying the full forms of neuron types present in this work.

References

[1] Christos Giatsidis, Dimitrios M Thilikos, and Michalis Vazirgiannis. D-cores: measuring collaboration of directed graphs based on degeneracy. *Knowledge and information systems*, 35(2): 311–343, 2013.

[2] Louis K Scheffer, C Shan Xu, Michal Januszewski, Zhiyuan Lu, Shin-ya Takemura, Kenneth J Hayworth, Gary B Huang, Kazunori Shinomiya, Jeremy Maitlin-Shepard, Stuart Berg, et al. A connectome and analysis of the adult drosophila central brain. *Elife*, 9:e57443, 2020.

[3] Réka Albert, Hawoong Jeong, and Albert-László Barabási. Error and attack tolerance of complex networks. *nature*, 406(6794):378–382, 2000.
